# Generalized chest CT and lab curves throughout the course of COVID-19

**DOI:** 10.1038/s41598-021-85694-5

**Published:** 2021-03-25

**Authors:** Michael T. Kassin, Nicole Varble, Maxime Blain, Sheng Xu, Evrim B. Turkbey, Stephanie Harmon, Dong Yang, Ziyue Xu, Holger Roth, Daguang Xu, Mona Flores, Amel Amalou, Kaiyun Sun, Sameer Kadri, Francesca Patella, Maurizio Cariati, Alice Scarabelli, Elvira Stellato, Anna Maria Ierardi, Gianpaolo Carrafiello, Peng An, Baris Turkbey, Bradford J. Wood

**Affiliations:** 1 Center for Interventional Oncology, Radiology and Imaging Sciences, NIH Clinical Center and National Cancer Institute, National Institutes of Health, Bethesda, MD 20892 USA; 2 Philips Research North America, Cambridge, MA 02141 USA; 3 Radiology and Imaging Sciences, NIH Clinical Center, National Institutes of Health, Bethesda, MD 20892-1182 USA; 4 National Cancer Institute, National Institutes of Health, Bethesda, MD 20892 USA; 5 Clinical Research Directorate, Frederick National Laboratory for Cancer Research, NCI, Frederick, MD 21702 USA; 6 NVIDIA Corporation, Bethesda, MD 20892 USA; 7 NVIDIA Corporation, Santa Clara, CA 95051 USA; 8 Division of International Epidemiology and Population Studies, Fogarty International Center, National Institutes of Health, Bethesda, MD 20892 USA; 9 Critical Care Medicine Department, NIH Clinical Center, National Institutes of Health, Bethesda, MD 20892 USA; 10 Diagnostic and Interventional Radiology Service, ASST Santi Paolo e Carlo, Milan, Italy; 11 Department of Radiology and Department of Health Sciences, Fondazione IRCCS Cà Granda Ospedale Maggiore Policlinico and University of Milano, 20122 Milan, Italy; 12 Postgraduate School of Diagnostic and Interventional Radiology, University of Milan, Milan, Italy; 13 Department of Radiology, Xiangyang NO. 1 People’s Hospital Affiliated to Hubei University of Medicine, Xiangyang, Hubei, 441000 China; 14 Molecular Imaging Branch, National Cancer Institute, National Institutes of Health, Bethesda, MD 20892 USA; 15 National Institute of Biomedical Imaging and Bioengineering, Bethesda, MD 20892 USA

**Keywords:** Outcomes research, Computed tomography, Three-dimensional imaging, Machine learning, Laboratory techniques and procedures

## Abstract

A better understanding of temporal relationships between chest CT and labs may provide a reference for disease severity over the disease course. Generalized curves of lung opacity volume and density over time can be used as standardized references from well before symptoms develop to over a month after recovery, when residual lung opacities remain. 739 patients with COVID-19 underwent CT and RT-PCR in an outbreak setting between January 21st and April 12th, 2020. 29 of 739 patients had serial exams (121 CTs and 279 laboratory measurements) over 50 ± 16 days, with an average of 4.2 sequential CTs each. Sequential volumes of total lung, overall opacity and opacity subtypes (ground glass opacity [GGO] and consolidation) were extracted using deep learning and manual segmentation. Generalized temporal curves of CT and laboratory measurements were correlated. Lung opacities appeared 3.4 ± 2.2 days prior to symptom onset. Opacity peaked 1 day after symptom onset. GGO onset was earlier and resolved later than consolidation. Lactate dehydrogenase, and C-reactive protein peaked earlier than procalcitonin and leukopenia. The temporal relationships of quantitative CT features and clinical labs have distinctive patterns and peaks in relation to symptom onset, which may inform early clinical course in patients with mild COVID-19 pneumonia, or may shed light upon chronic lung effects or mechanisms of medical countermeasures in clinical trials.

## Introduction

The role of chest Computed Tomography (CT) in the SARS-CoV-2 pandemic is highly dependent upon local practice patterns and available resources ^1^. Scarce data are available on the clinical or research utility of applying tools prospectively for quantitative metrics in patients presenting with pre-symptomatic coronavirus disease 2019 (COVID-19) opacities. The role of imaging in COVID-19 is ill-defined, perhaps in part due to a lack of widespread uniform use, which limits size and availability of data ^2^. Although rational, the World Health Organization (WHO) guidance for chest imaging for COVID-19 is based on expert opinion and low-certainty evidence. Additional evidence would thus be welcome regarding its clinical utility ^3^. The lower sensitivity of RT-PCR in early and pre-symptomatic disease as well as imperfect and variable turn around times highlights the potential impact of additional tests with immediate readouts, such as targeted CT in high risk exposed populations ^4^. The cohort presented here showed pre-symptomatic CT findings, which adds experiential evidence towards support of the feasibility of CT utility. Although no major therapeutic or vaccine clinical trials implement CT response criteria, a standardized CT quantification tool could possibly detect relevant signals of response or early disease modulation after medical countermeasures. Specifically, the number of affected lobes, extent of well aerated lung, or consolidation at baseline chest CT may predict outcomes in selected populations ^5,6^. Quantification of CT opacities mirrors clinical severity in patients with COVID-19 ^7–9^, however standardized, automated and validated assessment tools have not been widely applied to clinical practice nor to clinical trials of COVID-19 therapeutics.

Prior work has evaluated the temporal dynamics of CT features in patients with COVID-19, however, thus far has been focused on post-symptomatic timepoints ^10–13^. Far less is known about the CT appearance in the pre-symptomatic period, nor how this relates to subsequent overall course of the disease. Knowledge of the typical chronology and sequence of the lung opacity volumes and subtypes may inform treatment decisions. Further, deviations from expected patterns before or after symptom onset may carry prognostic or early treatment implications. Ground glass opacities (GGO) are the most common and typical feature on chest CT in patients with COVID-19, however, opacity type and pre-symptomatic CT findings remain incompletely defined ^13–15^.

Quantitative analysis of sequential CTs over time may characterize COVID-19 and better inform efforts to develop drugs or to assess response longitudinally over the course of a hospitalization or in chronic disease. In this study we aimed to evaluate the dynamics of chest CT opacity subtypes in COVID-19 before and after symptom onset. Additionally, the dynamic correlation of symptoms, laboratory data, and CT opacity metrics over the course of infection may provide a standardized reference.

## Results

### Descriptive CT findings

In total, 121 chest CTs were analyzed from 29 patients. Each patient had an average of 4.2 ± 1.5 CTs (range 2–8 CTs). COVID-19 related opacity was present in all patients at some point during infection and opacities were present in 87% of the CTs (105/121). The main opacity subtype encountered was GGO (84%, 102/121), followed by consolidation (59%, 71/121), atelectasis (27%, 33/121), sub-pleural reticulations (10%, 12/121), and crazy paving (7%, 8/121). Pleural effusion was noted in 10% (12/121).

The opacities were bilateral in 77% (81/105), diffuse upper and lower in 63% (66/105), inferior lobes only in 32% (34/105), and only superior lobes involvement in 4% (4/105). Opacities often had mainly a peripheral distribution (96%, 101/105) or less commonly central and peripheral distribution (7%, 7/105). The average number of distinct opacity foci was 3.9 (range 1–13).

**Figure 1:**
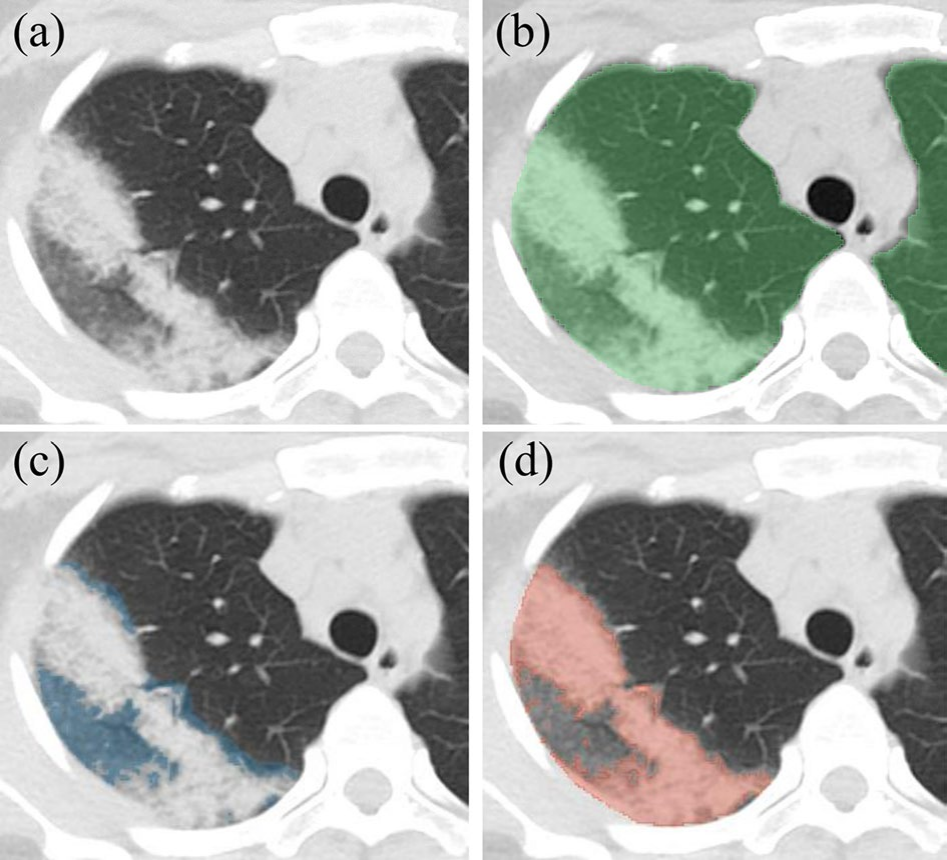
Axial CT images of COVID-19 opacity in right upper lobe, resulting in a part of GGO and a part of consolidation. (**a**) Non-contrast axial chest CT passing through right upper lobe opacity. (**b**) Overlaid AI whole lung segmentation (green). (**c**) Overlaid GGO segmentation alone (blue). (**d**) Overlaid consolidation segmentation alone (red).

Segmentations clearly and independently delineated total lung with AI, and overall opacity, GGO, and consolidation with manual segmentation (Fig. 1). The AI total lung segmentations were verified by radiologists as subjectively accurate, with no major inaccuracies identified (Fig. 1a).

### Dynamic curves for percent lung opacity

**Figure 2:**
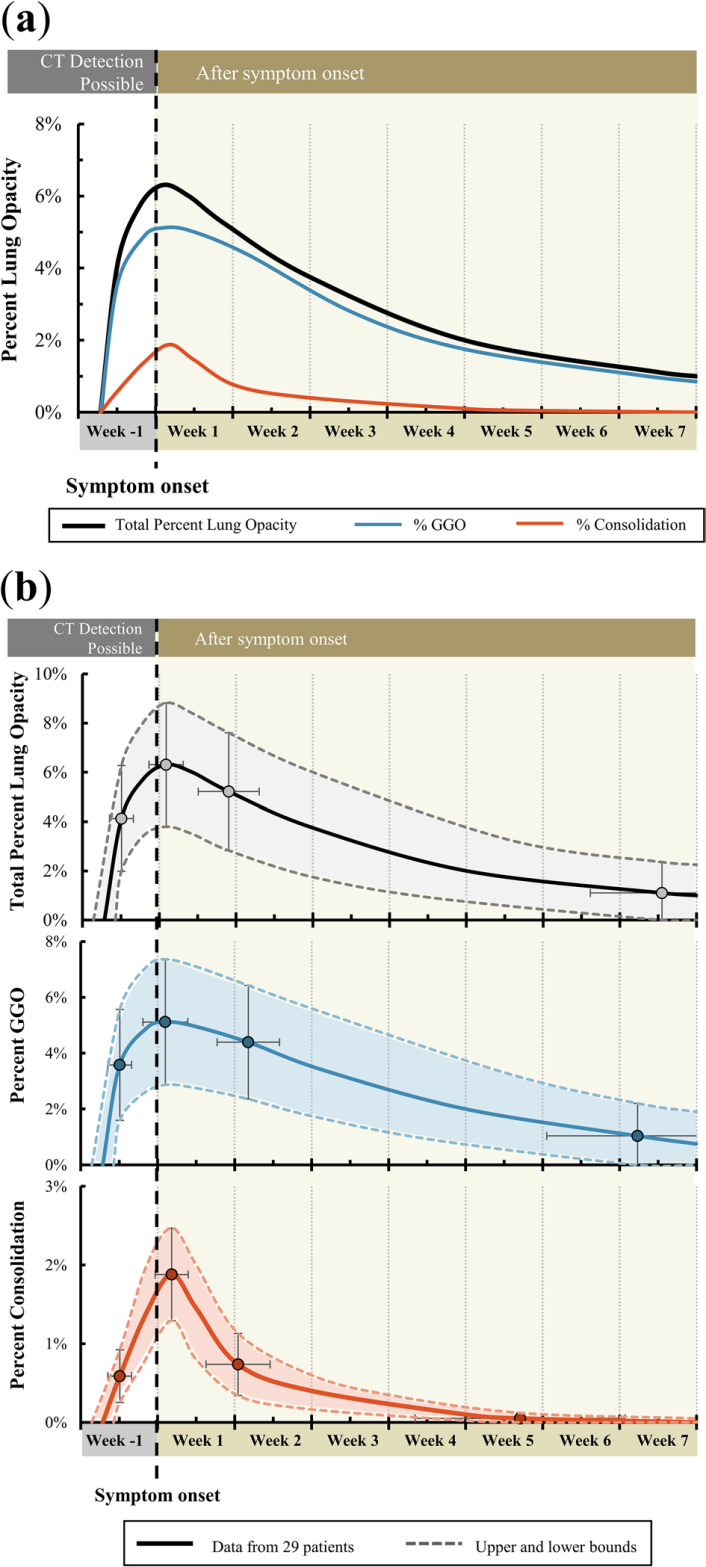
Dynamic curves of percent lung opacities for initially asymptomatic patients with COVID-19. (**a**) COVID-19 lung opacities, generalized from 29 patients with sequential CTs. Percent COVID-19 lung involvement (black) and opacity subtypes, including GGO (blue) and consolidation (red) are shown. (**b**) Detailed curves showing total percent COVID-19 lung opacity and subtype (GGO and consolidation). The upper and lower bounds and error bars represent the standard deviations. Data points are shown at the 4 generalized time points where percent lung involvement was calculated and estimated.

Dynamic curves for percent COVID-19 lung opacity in this population demonstrated the disease course on CT over time (Fig. 2, Table 1). Standard deviations are visually appreciated on detailed curves that define the range of the data with upper and lower bounds (Fig. 2b). Individual patient curves are displayed in Supplemental Figure S1. CT data resides in a public repository “The Cancer Imaging Archive” (https://www.cancerimagingarchive.net/).

**Table 1:** Data used to generate the dynamic curves for percent lung opacity.

		Day		% Lung involvement		# Of patients
		Average	SD	Average	SD	
Total % Lung opacity	First CT Scan	− 3.4	2.2	4.1	4.3	29
	Maximum Opacity	0.6	3.1	6.3	5.0	29
	Next CT after maximum opacity	6.3	5.6	5.2	4.8	18
	Minimum opacity/last CT	45.8	13.1	1.1	2.5	25
% GGO Lung involvement	First CT Scan	− 3.4	2.2	3.6	4.0	28
	Maximum % GGO	0.7	4.1	5.1	4.5	28
	Next Scan	8.2	5.7	4.4	4.1	17
	Minimum % GGO/last CT	43.6	16.5	1.0	2.3	25
% Consolidation Lung involvement	First CT Scan (with consolidation)	− 3.4	2.2	0.6	0.7	27
	Maximum % GGO	1.3	3.0	1.9	1.2	26
	Next Scan	7.3	5.8	0.7	0.8	16
	Minimum % GGO/last CT	33.0	19.1	0.1	0.1	23

Lung opacities were observed an average of 3.4 ± 2.2 days prior to symptom onset (maximum seven days). Both GGOs and consolidation were present on initial CT in all patients. A majority of lung opacity volume was classified as GGO (3.6 ± 0.4.0% of lung volume and 87% of all opacity volume).

**Figure 3:**
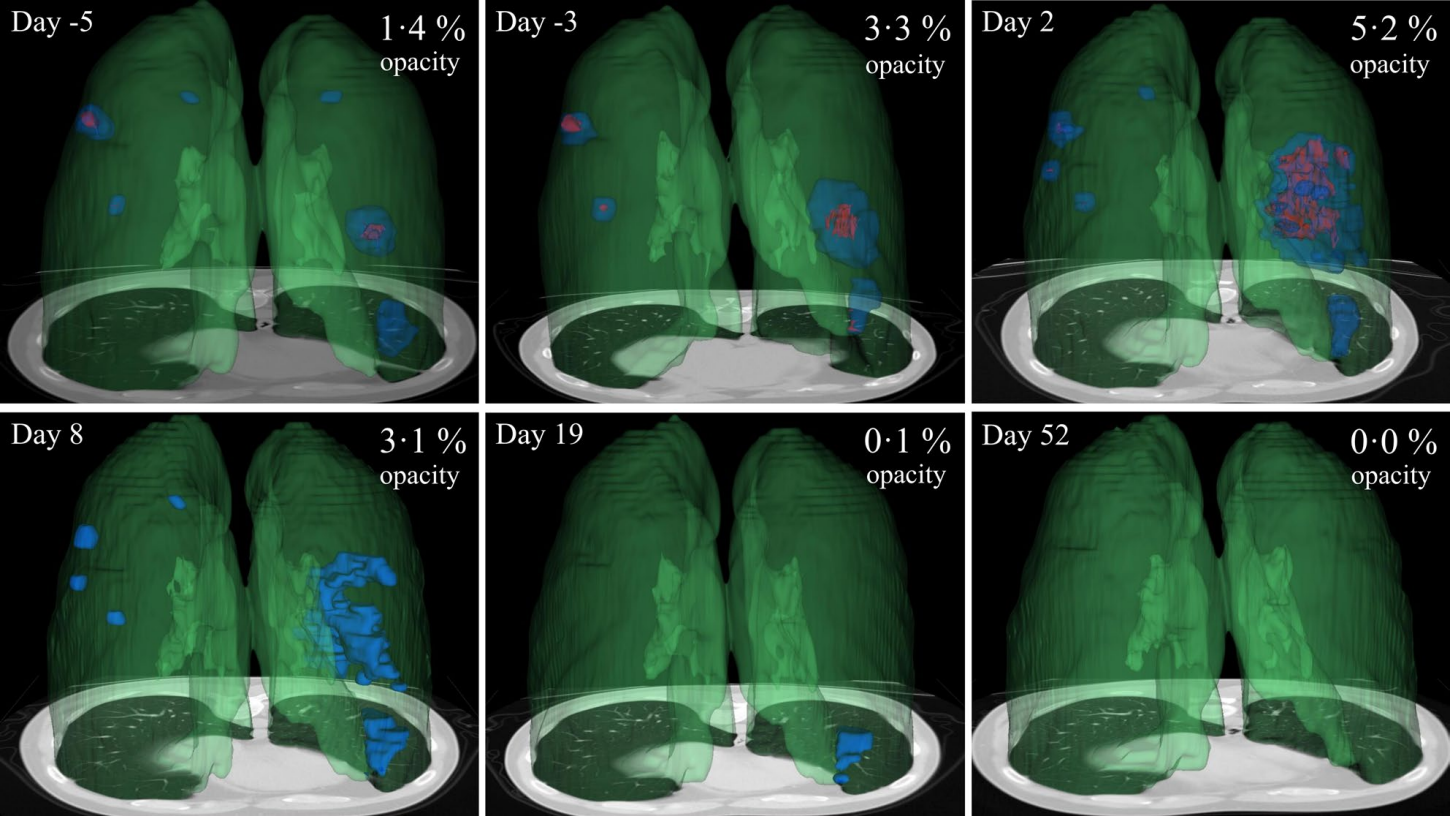
3D model display of the evolution of COVID-19 opacities of 34 years old male from pre-symptomatic stage to convalescence. Symptom onset is day 0. Green shows 3D whole lung segmentation (derived from an AI model), blue shows GGO lesions, and red shows consolidation. The opacity volume (blue plus red) divided by the whole lung volume (green) is the percent lung opacity. Note normal hilar anatomy (dark green).

The maximum percent lung involvement was found 0.6 ± 3.1 days after symptoms onset. Similarly, the maximum percent involvement of GGOs was found 0.7 ± 4.1 days after symptom onset. The maximum percent involvement of consolidation was found slightly later, at 1.3 ± 3.0 days after symptom onset. At its peak, percentage lung involvement was 6.3 ± 5.0%. Peak GGO and consolidation percentages were 5.1 ± 4.5% and 1.9 ± 1.2%, respectively. Whole lung and opacity subtype segmentations over time were reconstructed and visualized in Fig. 3.

Opacities (primarily GGO) persisted in a diminished fashion on the last CT (in 14 of 29 patients), with an overall percent lung opacity average of 1.1 ± 2.5% at an average of 46 ± 13 days after symptom onset. The percentage of GGO and consolidation involvement decreased by half of the peak 25 and 6 days later, respectively. At the last follow-up, GGOs accounted for 1.0 ± 2.3% of the lung 44 ± 17 days after symptom onset and consolidation accounted for 0.1 ± 0.1% at 33 ± 19 days after symptom onset.

### Dynamic curves for COVID-19 lesion attenuation

**Figure 4:**
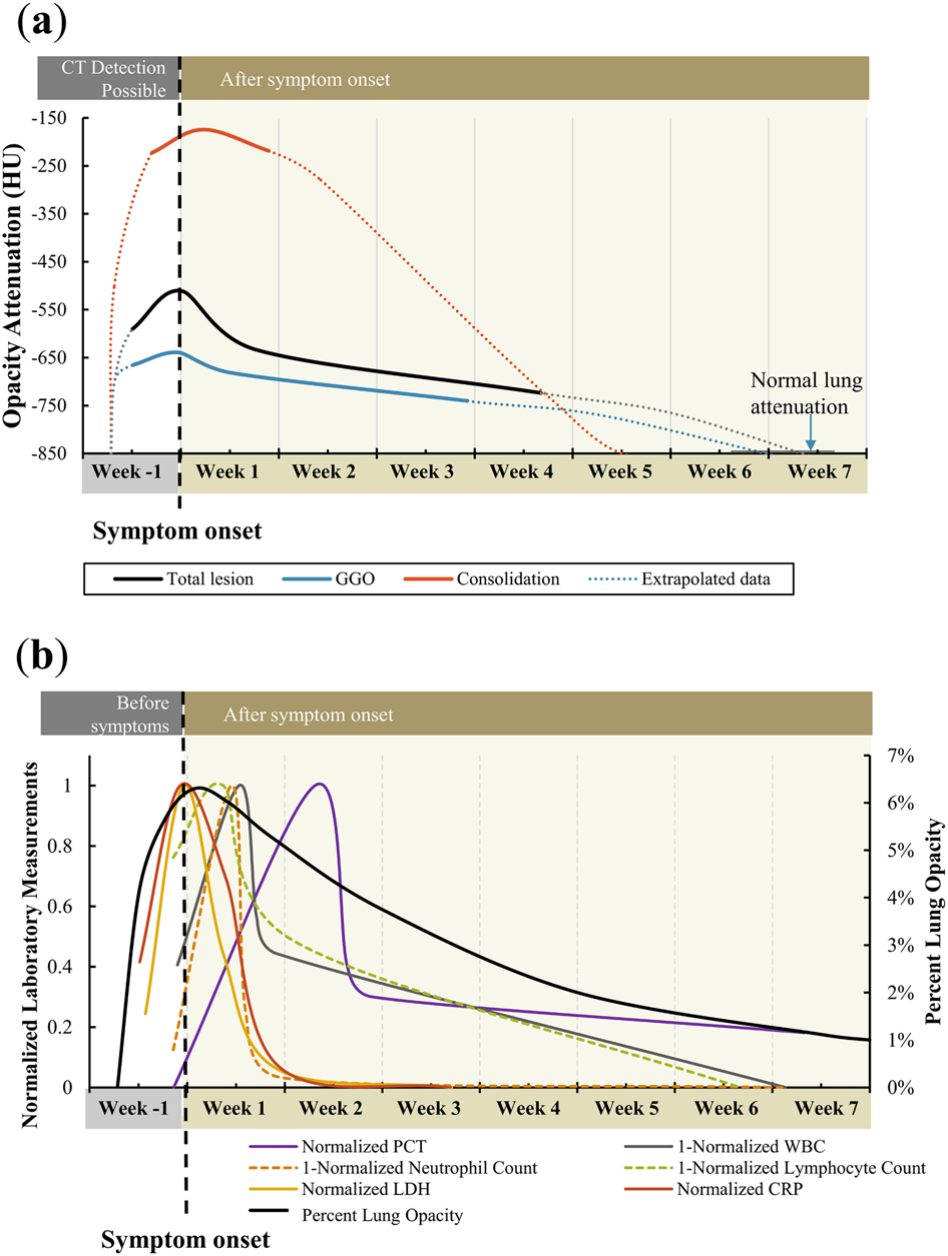
Dynamic curves of lesion attenuation and laboratory measurements. (**a**) Dynamic curves for COVID-19 lesion attenuation or density. The total lesion attenuation (black) and subtypes (GGO = blue, consolidation = red) are shown overtime and were generalized from 29 patients with sequential CTs. The dotted lines show the data extrapolated to normal lung attenuation. (**b**) Dynamic laboratory measurements over time for COVID-19-positive patients. The generalized and normalized curves for PCT (purple), 1-WBC (gray), LDH (yellow), CRP (red) with total percent lung involvement (black).

Dynamic curves for lesion attenuation during the disease time course were generated (Fig. 4a, Table 2). Standard deviations on detailed curves defined the range of the data with upper and lower bounds (Supplemental Figure S2). Data was extrapolated to the attenuation of the healthy lung (− 862 ± 56 HU). Only three data points were used to generate the dynamic consolidation attenuation curve as it was less prevalent. The maximum attenuation of the overall opacities occurred the same day of symptom onset (0.0 ± 3.1 days). The maximum GGO attenuation occurred just before symptom onset at − 0.2 ± 3.4 days, while consolidation attenuation peaked later at 1.6 ± 5.1 days.

**Table 2:** Data used to generate the dynamic curves for COVID-19 lesion attenuation.

		Day		Lesion attenuation (HU)		# Of patients
		Average	SD	Average	SD	
Overall opacity attenuation	First CT Scan	− 3.4	2.2	− 590	104	29
	Maximum Opacity	0.0	3.1	− 511	116	28
	Next CT after maximum opacity	5.4	4.1	− 634	108	15
	Minimum opacity/last CT	25.7	17.2	− 724	74	19
GGO attenuation	First CT Scan (with GGOs)	− 3.4	2.2	− 665	65	28
	Maximum % GGO	− 0.2	3.4	− 639	54	27
	Next Scan	3.6	4.0	− 682	62	17
	Minimum % GGO/last CT	20.5	15.9	− 741	54	19
Consolidation attenuation	First CT Scan (with consolidation)	− 2.1	3.1	− 223	52	27
	Maximum % GGO	1.6	5.1	− 174	50	21
	Minimum % consolidation	6.3	5.6	− 219	91	11

### Dynamic curves for laboratory measurements

From all laboratory measurements, procalcitonin (PCT), white blood cell count (WBC), lactate dehydrogenase (LDH), and c-reactive protein (CRP) were found to have similar dynamic curves to percent lung opacity (increase after infection, sharp peak, and decline) and were selected for demonstration of the curves. The average time between the first laboratory test to last laboratory test was 36 ± 22 days. In total, PCT and WBC were measured an average of 3.6 ± 1.3 times per patient. LDH and CRP were measured a total of 30 and 37 times respectively with an average of 1.2 ± 1.0 measurements per patient for both.

The normalized curves for PCT, WBC (inverted), LDH, and CRP over time were generated (Fig. 4b, source data Table 3).

**Table 3:** Data used to generate the dynamic curves for PCT, WBC, and neutrophil and lymphocyte count.

		Day		Value		# Of patients
		Average	SD	Average	SD	
PCT	First laboratory test	− 1.0	5.1	0.194	0.066	27
	Maximum PCT	9.2	10.6	0.295	0.061	28
	Next lab test after maximum	13.3	6.4	0.225	0.092	10
	Last follow-up lab test	44.3	15.7	0.213	0.094	17
WBC (× 10^9^/L)	First laboratory test	− 0.7	5.3	5.3	1.6	26
	Minimum WBC	3.7	7.5	4.1	1.3	27
	Next lab test after minimum	6.4	6.0	5.2	1.3	16
	Last follow-up lab test	42.9	14.6	6.2	1.8	19
Neutrophil count (× 10^9^/L)	First laboratory test	− 1.0	4.0	3.4	1.6	27
	Minimum neut. count	3.2	5.9	2.4	1.1	28
	Next lab test after minimum	6.8	5.6	3.5	1.5	18
	Last follow-up lab test	42.7	15.9	3.5	1.7	20
Lymphocyte count (× 10^9^/L)	First laboratory test	− 1.0	4.0	1.3	0.4	27
	Minimum lymph. count	2.6	6.2	1.1	7.5	28
	Next lab test after minimum	7.5	10.7	1.6	0.5	21
	Last follow-up lab test	39.7	18.1	2.1	0.7	22
LDH (mmol/L)	Early screen (day − 4 to − 2)	− 3.0	0.8	213	62	7
	Peak (day − 1 to 1)	− 0.3	1.0	314	104	4
	Follow-up 1 (day 2 to 4)	2.6	0.8	239	69	5
	Follow-up 2 (day 5 to 7)	6.4	0.6	189	26	5
	Follow-up 3 (day 8 +)	19.0	6.1	181	37	9
CRP (mg/L)	Early screen (day − 4 to − 2)	− 3.4	0.8	17.4	11.2	5
	Peak (day − 1 to 1)	− 0.5	0.8	40.8	30.4	6
	Follow-up 1 (day 2 to 4)	2.8	1.0	28.5	30.4	9
	Follow-up 2 (day 5 to 7)	6.5	0.6	3.7	2.8	6
	Follow-up 3 (day 8 +)	18.9	13.2	0.8	7.2	9

Standard deviations on detailed curves define the range of the data with upper and lower bounds (Supplemental Figure S3). The peaks of PCT and WBC (inverted) were seen 9.2 ± 10.6 and 4.1 ± 1.3 days respectively after symptom onset. In contrast, peak LDH and CRP occurred at symptom onset (at − 0.3 ± 1.0 days and − 0.5 ± 0.8 days, respectively). Peaks values for PCT, WBC, LDH, and CRP were 0.295 ± 0.061 ng/mL, 4.1 ± 1.3 × 10^9^/L, 314 ± 104 mmol/L, and 40.8 ± 30.4 mg/L, respectively. Minimum neutrophil and lymphocyte counts were 2.4 ± 1.1 × 10^9^/L at 3.2 ± 5.9 days and 1.1 ± 0.4 × 10^9^/L at 2.6 ± 6.2 days after symptom onset, respectively.

In this early disease cohort, CT opacities and certain labs colocalized and peaked very near the day of symptom onset. More sequential data points were available for WBC and PCT (average of 3.7 tests per patient), and therefore the same methodology for curve formation was applied as that for CT analysis. The dynamic curves of LDH and CRP however required data aggregation and pooling to extrapolate curves based upon pooling of different patients’ data, due to the limited data points available for individual patients (average 1.2 data point per patient). Therefore, 3-day increments were aggregated and averaged until the 10th day after symptom onset for the first points, and then all data points over 10 days were aggregated to derive the last point of the curve.

### Correlation analysis

**Figure 5:**
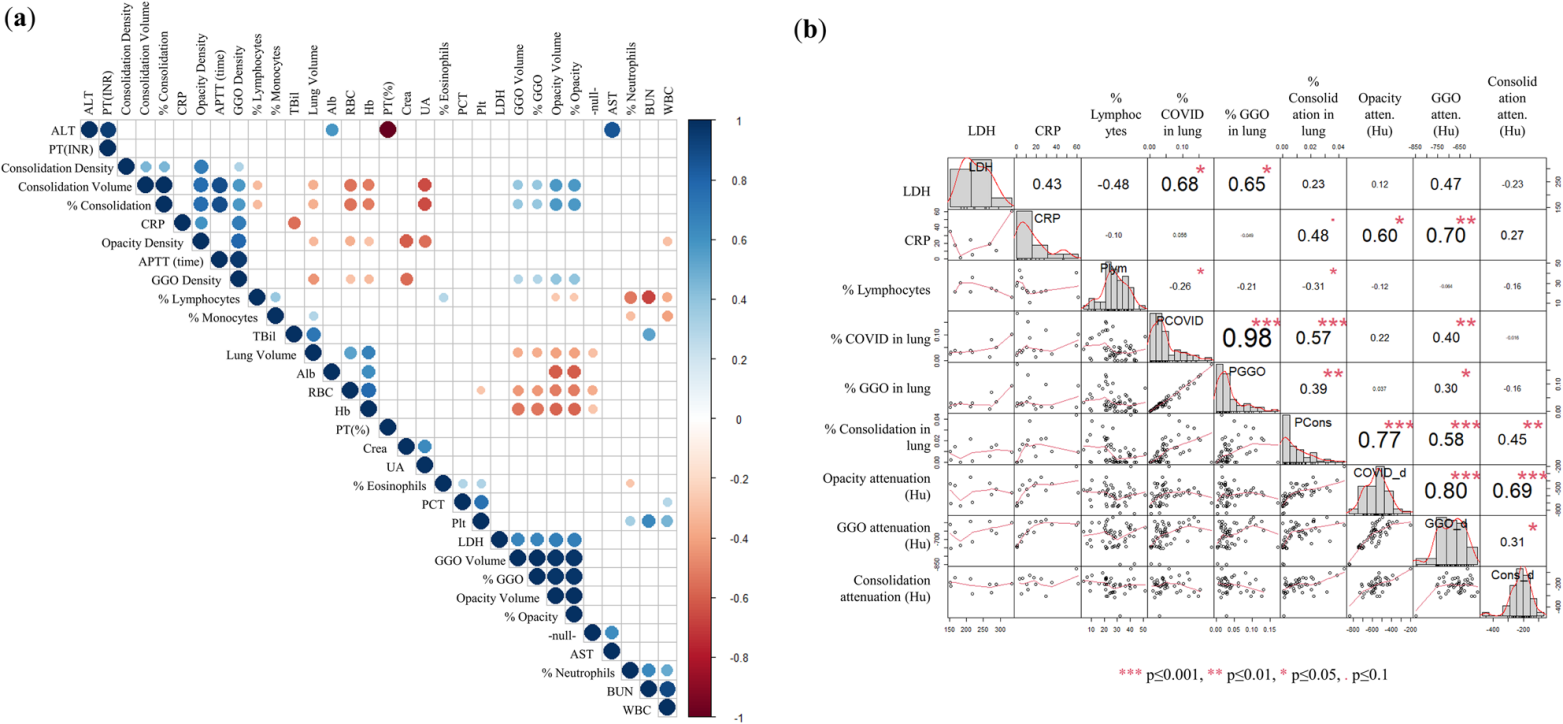
Correlation analysis between CT findings and laboratory measurements. (**a**) Correlation diagram heat map of all investigated CT features and laboratory measurements. Dots are present when p ≤ 0.05 and color represent either a positive (blue) or negative (red) correlation coefficient (r). (**b**) Detailed correlogram of features with strong correlation coefficients low probability coefficients (low p-values) .

A correlation analysis between CT findings and laboratory measurements found a strong correlation between LDH and percent lung opacity (r = 0.68, p = 0.03) and percent GGO (r = 0.65, p = 0.04), between CRP and opacity attenuation (r = 0.60, p = 0.01) and GGO attenuation (r = 0.70, p = 0.003), and between lymphocyte percentage and percent lung opacity (r = − 0.26, p = 0.05) and percent consolidation (r = − 0.31, p = 0.02). A correlation diagram heat map of all investigated CT features and laboratory measurements and a detailed correlogram of features with strong correlation coefficients low probability coefficients are given in Fig. 5.

### Referencing dynamic curve for percent lung opacity in one patient

**Figure 6:**
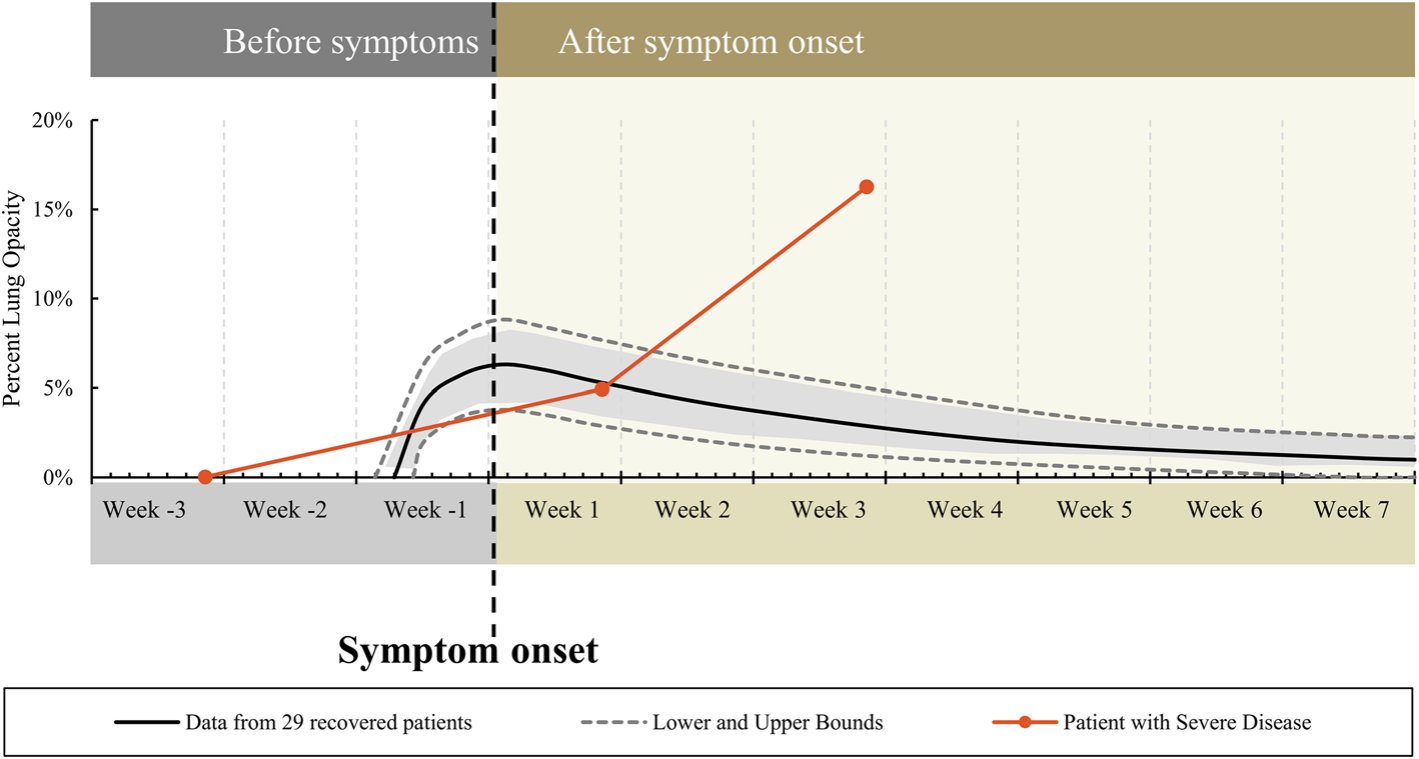
Demonstration of the utility of the dynamic curve using a pre-selected patient with severe COVID-19. Percent lung opacity was calculated from 3 serial chest CT’s. Deviation from the mild to moderate disease curve occurs 9 days after symptom onset, as a clinical "red flag".

Mapping a single pre-selected external example patient to the CT reference curve for mild COVID-19 disease demonstrated the feasibility of using the dynamic curve as a clinical reference tool to display clinical course severity over time (Fig. 6). Deviation of the CT percent opacity from the reference curve for mild disease is shown in one example, with limited significance. This applied example of pre-selected severe disease in an independent sight-unseen demographic within another outbreak setting is displayed as a deviation from the generalized curve for mild to moderate disease. External validation with correlative outcomes was not performed. An applied example is presented for dynamic lab and imaging referencing to generalized curves, to show theoretical applied use of such curves (Supplemental video).

## Discussion

Clearer definition of the clinical manifestations of COVID-19 over time could provide an essential reference point and measurement system for future applied studies. CT opacities pre-dated symptom onset in COVID-19 by an average of three days in this highly selected cohort of early disease in a high prevalence outbreak setting. Opacity volume, subtype and attenuation curves follow a parallel pattern to key clinical and laboratory factors ^16^. In this study, the high number of CT scans per patient (average = 4.2) adds strength to the sequential CT temporal analysis model. Prior serial CT analyses have generally not included > 2 multiple CTs in the same patient, as seen here. A reference curve built upon same-patient data is useful to patients with even just one CT at various timepoints in the disease course. In June 2020 the World Health Organization (WHO) clarified indications for imaging as when: PCR is unavailable or delayed, PCR is negative but with a high index of suspicion, patients at high risk, over 60 years old, or with comorbidities, for triage to ward or ICU, hospitalized patients who progress or are unresponsive to therapies, as well as patients with suspicion for pulmonary embolus or pulmonary fibrosis ^3^. This represented an expansion of indications, compared to previous guidelines, enhancing the potential utility of a generalized reference to define an expected or mild disease course ^1^.

Standardized COVID-19 quantification of chest CT opacities may non-invasively and rapidly characterize disease, which may be valuable to investigate as one element of a composite outcome measure or predictive surrogate endpoint in clinical trials. Sequential CT data presented here informs a generalized dynamic curve that may provide a useful reference for specific patients in a similar setting. Standardization by date of symptom onset allows comparison and characterization of patients who may not yet have declared themselves along a specific disease trajectory. Such comparisons to a generalized curve could theoretically help define risk, triage, resource allocation, or need for timely early medical countermeasures upon deviation from an expected normalized curve for similar patients. Such curves could also clarify presence and degree of chronic lung opacity, which could help decipher chronic lung effects. Residual opacities averaging 1.1% were present at last CT scan (average of 46 days), which raise the concern for residual pulmonary impairment after convalescence, and certainly merit further investigation of chronic effects. Chest CT data in a research or clinical trial setting documents actual lung disease that might be otherwise hidden by clinical trials that only follows secondary effects and externally apparent clinical metrics.

GGOs are known to be the primary lung opacity in COVID-19. In the studied cohorts, consolidation peaked later in the patient course and resolved faster than GGO. Consolidation however is associated with a poorer outcome ^6^. This suggests mucous or debris within the alveolar air sacs associated with SARS-CoV-2 may evolve over the course of the infection and this change may be detected with CT. Residual opacity or scarring on CT persisted during convalescence an average of 46 days after symptom onset, which is of unknown significance. Future studies merit evaluation of outcomes associated with consolidation or other combination of patterns, and the potential predictive role for CT AI in triaging into specific treatment tracks.

The model used a combination of manual and automated deep learning segmentation. The segmentation of opacities was accomplished manually, whereas whole lung segmentation was achieved via a deep learning model. Indeed, manual whole lung segmentation in diseased lungs is very time consuming and impractical for radiologists, however almost instantaneous with deep learning artificial intelligence ^8^. Quantitative CT has been studied in COVID-19 as a tool for triage, disease progression and severity, outcome prediction, drug discovery, and laboratory test correlation ^7,8,17–21^. Any quantification tool applied over time adds another dimension of information to the evolving knowledge base in this viral pandemic.

With contextual and temporal referencing, CT data may define when and how a patient’s disease course might vary or diverge from that expected for an uncomplicated recovery (Fig. 6). CT could potentially also play an enhanced role in clinical trials by defining reproducible response criteria for triage, prognostication, or clinical alerts in the context of comparison to a standard dynamic curve or “reference nomogram”. The speculative potential for such a tool may support development of a quantification tool as a part of the evaluation of similarly infected patients (with comparable variables of community prevalence, age, underlying lung disease or comorbidities, etc.).

Symptom and RT-PCR screening remain key metrics for re-opening or back to work (or school) strategies ^22^. The potential role of imaging has evolved over the course of the pandemic, however no consensus exists on exact utility. In the event that RT-PCR testing is unavailable, delayed, or clinically suspected to be false negative, then imaging may play a role in the diagnosis of COVID-19 ^3^. The WHO suggests not using chest imaging for diagnosis in asymptomatic contacts of patients with COVID-19 ^3^ However, 27 of 29 patients here with contact or travel history had CT opacities averaging 3.4 days before symptom onset. Also, given the recent confusion over testing in the asymptomatic population ^23^, the fact that chest CT here showed infiltrates before symptoms developed merits further consideration. This is especially relevant, given the unmet need for evidence to guide testing and imaging recommendations that are currently rated as low level of evidence, resulting in skepticism, heterogeneity of practice, and confusion ^3^.

The dataset presented here was somewhat atypical, in that almost all patients received CT and laboratory tests before symptoms developed. The poorly defined pre-symptomatic phase of the SARS-CoV-2 infections was explored here with CT and laboratory findings. Prior studies with CT data have pooled intervals and patients in order to generate curve estimates. In comparison, the present study utilizes multiple sequential CTs from the same patient in order to generate a higher fidelity dynamic curve, without the screening bias present from pooling data among patients.

Prior case reports have attempted to define patient-specific curves with serial CT ^24^. Pan et al. used a CT score based on the sum of each lobe score calculated from a range of percent involvement, and therefore grouped patients with different severity. In addition, their survivor group presented a great heterogeneity in patients’ severity with patients presenting CT severity score from 0 to 20, We feel that the use of total lung involvement percent might be more objective and reproducible way to describe severity, and less subject to reader subjectivity ^25^. Peak CT opacities on the day of symptom onset is a markedly different finding than seen on previous studies, one of which reported a peak CT opacity 10 days post-symptom onset ^8,11,12,26^.One possible explanation is patient selection bias. The cohort presented here may be relatively healthy with early disease and early CT targeted testing practices, in a high prevalence outbreak setting, perhaps with light viral exposure loads, although speculative. The extent of disease may be correlated with the viral exposure levels and times ^27^, thus it is possible that other reports describe patients who are sicker, presented a higher viral load or simply were selected for CT only when they became increasingly ill. This last bias speaks to the variability in the timing of CT either early alongside RT-PCR, versus later as a critical care tool, for patients who do not improve in expected timeframes ^26^. Our cohort included patients with mild to moderate initially asymptomatic pneumonia, where none required later ICU admission or intubation. Also, the average number of CT per patient was 4.2 in our study versus less than two for Wang et al. ^26^. More data points per patient is more internally controlled, thus less subject to model bias. In our study, patients served as their own controls, while in previous sequential studies, the evolution of opacity from symptom onset relied upon averaging the values within different time intervals. A supplemental analysis that compares previously reported methods to the present one is given in Supplemental Figure S4. These differences in methods dilutes individual patient dynamics and may explain why peak disease date is reported to be different in other studies.

Data aggregation and pooling certainly limit the reproducibility and reliability of those curves. Labs peaked close to the onset of symptoms and colocalized with peak opacities. Even though this methodology was based on only a few longitudinal datasets (8/20 for LDH), it has been widely used previously to display this type of sparse data ^10^.

This study is further limited by its retrospective nature and multiple major patient selection biases, which may preclude extrapolation to other patient populations. The total number of CTs (n = 121) and sequential laboratory tests (n = 105) is substantive, however 29 patients is a relatively small study. This single center study in a high prevalence setting limits generalizability or extrapolation to dissimilar or heterogeneous demographics. CTs were also not taken at standard times or uniform intervals with reference to symptom onset, and 2/29 had no CT before symptom onset. Awareness of outbreak and exposures may have lowered the threshold for presentation. The dynamic curves contain several underlying assumptions about the normalization of the data over time in order to construct a curve with intermediate points. Backfitting the pre-symptomatic curves to match a known pre-symptomatic normal/zero disease is somewhat arbitrary, based upon historical average serial interval of SARS-CoV-2, which could make the pre-symptomatic curve unreliable when applied as a reference. The segmentation process focused on attenuation thresholds which separated consolidation from ground glass segmentations and included small intra-parenchymal vessels in both. The effect was not analyzed independently.

Clinical utility of the laboratory curves remains undefined. However, it is possible although speculative that the dynamic radiographic and laboratory curves together might be useful as reference for clinical triage for borderline patients as an extra factor in support of increased level of acute care (Supplemental video). Dynamic reference curves may inform the relative disease status of positive SARS-CoV-2 patients, in relation to the expected course of mild disease. For example, if a patient’s CT data point resides within the dynamic reference curves, then mild disease may be likely, and the patient might be expected to improve during convalescence. In this way, a derived percent lung opacity on CT is a window into the clinical course, with severity of disease expressed as graphical deviation from a generalized curve. Although speculative, if the CT data point maps well outside the reference curve, then the patient deviating from mild disease might require consideration of more advanced or critical care. One example patient was selected for having advanced disease and serial chest CTs, and was plotted in comparison to the generalized curve for opacities, in order to show this point. The disease course in this example case demonstrates how the reference curves might function to define deviation, in a similar fashion to plotting an infant’s or toddler’s height and weight percentile to identify deviations from normal (Fig. 6). This single pre-selected case was specifically chosen to demonstrate the ability of the reference curves to alert for deviation from mild disease. However, this work without further prospective studies does not prove anything besides feasibility.

In conclusion, COVID-19 opacities were observed multiple days prior to symptom onset, culminated on the day of symptom onset and slowly decreased during follow-up in an initially asymptomatic cohort with early and mild to moderate COVID-19. Compared to GGOs, consolidation peaked later and almost resolved quicker in this cohort. Metrics for comparison could provide valuable clinical reference data for early detection of deviation from the expected disease course, which could potentially inform early medical countermeasures, therapeutic decision making, or clinical trial response criteria. Characterization of correlative patterns in such readouts can potentially identify patients with COVID-19 pneumonia by defining extreme deviations from standardized curves for patients with mild disease. Certainly a better understanding of serial disease changes on CT and labs over time could enlighten healthcare decision-makers facing off against an incompletely understood and novel virus in the context of medical countermeasures, chronic lung effects, or mutational variants.

## Methods

### Subjects

Xiangyang NO.1 People’s Hospital Affiliated to Hubei University of Medicine, Xiangyang, Hubei, China (Approval #20200702150947) and University of Milan (Universita Degli Studi Di Milano) Research Board/Institutional Review Board (IRB), Milan, Italy (#324-2020 and 562-2020 and #335-2020), provided approval for this study. The study was conducted in accordance with the local IRB and ethics approval for retrospective evaluation and data sharing. Informed consent requirements were waived by both Xiangyang No. 1 People’s Hospital Affiliated to Hubei University of Medicine, Xiangyang, Hubei, China (Approval #20200702150947) and University of Milan (Universita Degli Studi Di Milano) Research Board/IRB, Milan, Italy (#324-2020 and 562-2020 and #335-2020), due to the nature of the retrospective observational study.

Patients were retrospectively selected from a larger cohort of 739 patients who underwent initial CT screening alongside RT-PCR on the day of presentation at point of care settings in Hubei Province, China. 710 patients had only one CT. 29 patients who received 2 or more chest CTs over the course of their care during the initial hit phase of the pandemic were identified and analyzed for this study. All patients were RT-PCR positive for SARS-CoV-2. CT and laboratory tests were conducted between January 21, and April 12, 2020.

All 29 patients underwent screening with chest CT either because of a history of contact with patients with proven or suspected COVID-19 or because of high exposure risk due to travel in high prevalence regions (outbreak zones). The patients were predominately female (69%, 20/29), and were 41 ± 10 years old (range 25 to 60 years old). The hospitalization period was 15 ± 4 days and the symptomatic period was 10 ± 2 days. The overall follow-up period, defined as the time from first CT or laboratory measurement to last CT or laboratory measurement, was 50 ± 16 days (range 6 to 69 days). Demographic data, symptoms, and follow-up period is given in Table 4. The day of symptom onset was defined as day 0. Symptom onset was defined as the presence of fever (body temperature ≥ 37.8 °C) and one or more of the following: fatigue, headache, nasal discharge, sore throat, cough, myalgia, diarrhea, nausea or vomiting. Twenty-seven of 29 patients had a CT prior to symptom onset. No patients had a poor outcome or advanced disease requiring intervention. None of the 29 patients had a poor outcome or required intervention throughout the course of disease.

**Table 4:** Demographic data and follow-up periods for patients with sequential CTs and COVID-19.

	Number		Percent		
Female sex	20		69		
Chinese nationality	29		100		
Smoker	17		59		
Contact or suspected contact with COVID-19 Pt	29		100		
**Most common symptoms**					
Fever (temperature ≥ 37.8 °C)	7		24		
Fatigue	3		10		
Myalgia	3		10		
Nasal discharge or obstruction	3		10		
Sore throat	3		10		
Cough	3		10		

	Average	SD	Min	Max	Total
Age, years	41	10	25	60	
Symptomatic period, days	15	4	10	29	
Number of CTs	4.2	1.6	2	8	121
Number of lab tests	4.4	1.6	1	7	129
Total follow-up, days	50	16	6	69	
CT follow-up range, days	44	20	2	69	
Lab follow-up range, days	32	23	0	69	

### CT acquisition

Non-contrast chest CT was obtained with 120 kVp (Toshiba and GE Healthcare). The scans were reconstructed as axial images with 0.873 × 0.873 mm pixel size and 512 × 512 matrix with a standard slice thickness of 5 mm.

### CT interpretation

For each patient, initial chest CT was obtained on the day of presentation and subsequent sequential CT scans were obtained in intervals during a follow-up of 2 to 69 days after the initial scan (average 43 ± 20 days). Two radiologists, blinded to the clinical and laboratory data, retrospectively and independently reviewed each CT for the presence and location of opacities, type of opacity (GGO, consolidation, intralobular septal lines [“crazy paving”]), atelectasis, sub-pleural reticulation, mosaic attenuation, number of distinct opacities, upper/lower and peripheral/central location of the opacities, presence of pleural effusion, bronchial wall thickening, tree-in-bud nodules, and pre-existing lung disease (including emphysema, bronchiectasis, and fibrosis). All discrepancies were resolved by consensus review (174 out of 1815 entries).

Three radiologists manually annotated and segmented lung opacities (www.itksnap.org, www.slicer.org), and differentiated opacity subtypes of GGO and consolidation. Semi-automated (part manual) or assisted segmentation was based on a Hounsfield Unit (HU) attenuation threshold initially, which was subsequently refined manually. The optimal threshold was manually defined between − 400 and − 300 HU ^28–32^. Volume and average density were extracted for: (1) overall opacity, (2) GGO alone, and (3) consolidation alone. Other opacities listed in “CT Findings” including atelectasis and subpleural reticulations, contributed to overall opacities but were not independently segmented. Healthy lung was segmented using a manual threshold set between − 750 and − 650 HU, in order to extract the normal lung.

### Deep learning model

All chest CTs underwent automated whole lung segmentation using AI tools based on deep learning via a deep neural network model. The lung segmentation model was trained using a previously described model based upon an AH-Net architecture ^33^, and is publicly available as a part of the NVIDIA Clara Train SDK on NGC (NVIDIA Clara Train SDK v3 2020: https://docs.nvidia.com/clara/tlt-mi/clara-train-sdk-v3.0/index.html#). The extent of lung involvement was determined by combining manual and AI-based segmentation. Opacity volume (segmented manually) was divided by the overall lung volumes (segmented by AI model) to calculate percent lung involvement or percent COVID-19. AI-derived total lung segmentations were reviewed and verified by two radiologists.

### Dynamic curves of COVID-19 percent lung opacity and opacity attenuation

Dynamic curves for percent COVID-19 lung opacity and opacity attenuation were generated using a maximum of 4 points, including: (1) the first pre-symptomatic CT, (2) the maximum percent opacity/lesion attenuation, (3) the next subsequent CT, and (4) the last follow-up CT. These timepoints reflect relevant points during the disease progression, including: (1) initial point of care at a timepoint nearest to the first suspected risk event such as travel or contact with COVID-19 infected individual, (2) near or at symptom onset, (3) one week after symptom onset, (4) approximately one month after convalesce. The generalized function over time was created using a smoothed polynomial curve between points. The number of patients that were included in the generation of the curves is noted in the tables. To make the curve continuous, the day of infection was assumed to be 5 days prior to the known day of symptom onset ^27^.

### Laboratory data

All laboratory tests were assessed at admission or early in the disease and, for some measurements, also during follow-up between 1 and 69 days after initial measurements (average 36 ± 22 days). Measurements included 20 different labs: Blood Urea Nitrogen, Creatinine, Aspartate transaminase, Alanine transaminase, Bilirubin, Lactate Dehydrogenase (LDH), Albumin (Alb), C-reactive Protein (CRP), Uric Acid (UA), Procalcitonin (PCT), Red Blood Cell (RBC), Hemoglobin (Hb), Platelet (Plt), White Blood Cell (WBC), Neutrophil, Lymphocyte, Monocyte, Eosinophil percentage and count, Activated Partial Thromboplastin Time (APTT), and Prothrombin time and International Normalized Ratio (PT(INT)). 279 individual lab measurements were analyzed during the course of disease in the 29 patients. Twenty-six of 29 patients had laboratory tests prior to symptom onset.

### Dynamic curves of laboratory data

We analyzed all 20 laboratory measurements and identified PCT, WBC, LDH, and CRP for modeled curves. These dynamic curves for laboratory measurements were done in 2 fashions. For laboratory measurements that were taken an average of 3 or more times per patient during follow-up (PCT and WBC), laboratory curves were built similar to the opacity curves. Up to 4 data points were similarly defined, including: (1) the first laboratory measurement (2) the laboratory measurement of maximum or minimum value (depending on the trend), (3) the next subsequent laboratory measurement, (4) the last follow-up laboratory measurement.

In addition, for measurements that had two or fewer tests per patient (LDH and CRP), data were aggregated and averaged in 3-day increments until the 10th day after symptom onset. For these data, the curve was built from average laboratory values from the following days: (1) early screening: day − 4 to − 2, (2) at symptom onset: day − 1 to 1, (3) follow-up 1: day 2 to 4, (4) second follow-up: day 4 to 6, 5) third follow-up: day 8 and greater.

#### Correlation analysis

To explore the potential relationship between quantitative CT findings and laboratory test results, a Pearson’s correlation analysis was performed. The analysis was performed with CT findings and laboratory tests that were taken on the same day or one day apart.

### Example of referencing to dynamic curve for percent lung opacity

To demonstrate potential applied clinical utility of these curves, 1 patient from a different country with advanced disease was arbitrarily selected retrospectively from a separate external dataset of patients with SARS-CoV-2 positive PCR who had undergone 3 serial CTs during a severe disease course. This single patient who developed advanced severe disease was retrospectively mapped to, and plotted on, the generalized dynamic reference curve for lung opacity percent over time for patients with mild to moderate disease (Fig. 6).

## Supplementary Information

Supplementary Information 1.

Supplementary Video.

## Abbreviations

Alb: Albumin
ALT: Alanine aminotransferase
APTT: Activated partial thromboplastin time
AST: Aspartate aminotransferase
BUN: Blood urea nitrogen
CRP: C-reactive protein
GGO: Ground-glass opacity
Hb: Hemoglobin
LDH: Lactate dehydrogenase
PCT: Procalcitonin
Plt: Platelet
PT: Prothrombin time
PT(INR): Prothrombin time and international normalized ratio
RBC: Red blood cell count
TBil: Total bilirubin
UA: Uric acid
WBC: White blood cell count
