# Supplementary material for: Generalized chest CT and lab curves throughout the course of COVID-19
Source: Sci Rep. 2021 Mar 25;11:6940. doi: 10.1038/s41598-021-85694-5 (PMC7994835; doi:10.1038/s41598-021-85694-5)
Supplement: Supplementary file 1 — Supplementary Information 1. [file 41598_2021_85694_MOESM1_ESM.docx]

**Supplemental Material- Revision 2**

**Generalized Chest CT and Lab Curves Throughout the Course of COVID-19**

**Michael T. Kassin*, MD**

Center for Interventional Oncology, National Institutes of Health, Bethesda, MD, USA, 20892

Email: [michael.kassin@nih.gov](mailto:michael.kassin@nih.gov)

**Nicole Varble*, PhD**

Center for Interventional Oncology, National Institutes of Health, Bethesda, MD, USA, 20892

Philips Research North America, Cambridge, MA, USA, 02141

Email: [nicole.varble@nih.gov](mailto:nicole.varble@nih.gov)

**Maxime Blain*, MD**

Center for Interventional Oncology, National Institutes of Health, Bethesda, MD, USA, 20892

Email: [maxime.blain@aphp.fr](mailto:maxime.blain@aphp.fr)

**Sheng Xu, PhD**

Center for Interventional Oncology, National Institutes of Health, Bethesda, MD, USA, 20892

Email: [xus2@cc.nih.gov](mailto:xus2@cc.nih.gov)

**Evrim B. Turkbey, MD**

Department of Radiology and Imaging Sciences, National Institutes of Health, Bethesda, MD, USA, 20892

Email: [evrim.turkbey@nih.gov](mailto:evrim.turkbey@nih.gov)

**Stephanie Harmon, PhD**

National Cancer Institute, National Institutes of Health, Bethesda, MD, USA, 20892

Clinical Research Directorate, Frederick National Laboratory for Cancer, National Cancer Institute. Frederick, MD, USA, 21702

Email: [stephanie.harmon@nih.gov](mailto:stephanie.harmon@nih.gov)

**Dong Yang, PhD**

NVIDIA Corporation, Bethesda, MD, USA, 20892

Email: [dongy@nvidia.com](mailto:dongy@nvidia.com)

**Ziyue Xu, PhD**

NVIDIA Corporation, Bethesda, MD, USA, 20892

Email: [ziyuex@nvidia.com](mailto:ziyuex@nvidia.com)

**Holger Roth, PhD**

NVIDIA Corporation, Bethesda, MD, USA, 20892

Email: [hroth@nvidia.com](mailto:hroth@nvidia.com)

**Daguang Xu, PhD**

NVIDIA Corporation, Bethesda, MD, USA, 20892

Email: [daguangx@nvidia.com](mailto:daguangx@nvidia.com)

**Mona Flores, MD**

NVIDIA Corporation, Santa Clara, CA, USA, 95051

Email: [mflores@nvidia.com](mailto:mflores@nvidia.com)

**Amel Amalou, MS**

Center for Interventional Oncology, National Institutes of Health, Bethesda, MD, USA, 20892

Email: [amel.amalou@nih.gov](mailto:amel.amalou@nih.gov)

**Kaiyun Sun, PhD**

Division of International Epidemiology and Population Studies, Fogarty International Center, National Institutes of Health, Bethesda, MD, USA, 20892

Email: [kaiyuan.sun@nih.gov](mailto:kaiyuan.sun@nih.gov)

**Sameer Kadri, MD**

Critical Care Medicine Department, Clinical Center, National Institutes of Health, Bethesda, MD, USA, 20892

Email: [sameer.kadri@nih.gov](mailto:sameer.kadri@nih.gov)

**Francesca Patella, MD**

Department of Health Sciences, University of Milano, Italy

Department of Radiology, Fondazione IRCCS Cà Granda Ospedale Maggiore Policlinico, 10, 20122, Milano, Italy

Email: [battellina@gmail.com](mailto:battellina@gmail.com)

**Maurizio Cariati, MD**

Department of Health Sciences, University of Milano, Italy

Department of Radiology, Fondazione IRCCS Cà Granda Ospedale Maggiore Policlinico, 10, 20122, Milan, Italy

Email: [maurizio.cariati@asst-santipaolocarlo.it](mailto:maurizio.cariati@asst-santipaolocarlo.it)

**Alice Scarabelli, MD**

Postgraduate School of Diagnostic and Interventional Radiology

University of Milan

Milan, Italy

Email: [alice.scarabelli1105@gmail.com](mailto:alice.scarabelli1105@gmail.com)

**Elvira Stellato, MD**

Postgraduate School of Diagnostic and Interventional Radiology

University of Milan

Milan, Italy

Email: [Elvira.stellato@gmail.com](mailto:Elvira.stellato@gmail.com)

**Anna Maria Ierardi, MD**

Radiology Department

Fondazione IRCCS Cà Granda Ospedale Maggiore Policlinico

Milan, Italy

Email: [amierardi@yahoo.it](mailto:amierardi@yahoo.it)

**Gianpaolo Carrafiello, MD**

Radiology Department

Fondazione IRCCS Cà Granda Ospedale Maggiore Policlinico

Milan, Italy

Email: [gianpaolo.carrafiello@policlinico.mi.it](mailto:gianpaolo.carrafiello@policlinico.mi.it)

**Peng An, MD**

Department of Radiology, Xiangyang NO. 1 People’s Hospital Affiliated to Hubei University of Medicine, Xiangyang, 441000, China

Email: [lianxyyy2015@163.com](mailto:lianxyyy2015@163.com)

**Baris Turkbey, MD**

Molecular Imaging Branch, National Institutes of Health, Bethesda, MD, USA, 20892

National Cancer Institute, National Institutes of Health, Bethesda, MD, USA, 20892

Email: [ismail.turkbey@nih.gov](mailto:ismail.turkbey@nih.gov)

**Bradford J. Wood, MD**^†^

Center for Interventional Oncology, National Institutes of Health, Bethesda, MD, USA, 20892

Department of Radiology and Imaging Sciences, National Institutes of Health, Bethesda, MD, USA, 20892

National Cancer Institute, National Institutes of Health, Bethesda, MD, USA, 20892

National Institute of Biomedical Imaging and Bioengineering, Bethesda, MD, USA, 20892

Email: [bwood@nih.gov](mailto:bwood@nih.gov)

Phone: 301-728-0362

*Contributed equally to manuscript

^†^Corresponding Author:

Bradford J. Wood

[bwood@nih.gov](mailto:bwood@nih.gov)

301-728-0362

Radiology & Imaging Sciences / Clinical Center, National Institutes of Health

MSC 1182, Bldg. 10, Room 1C341, Bethesda, MD 20892-1182, USA


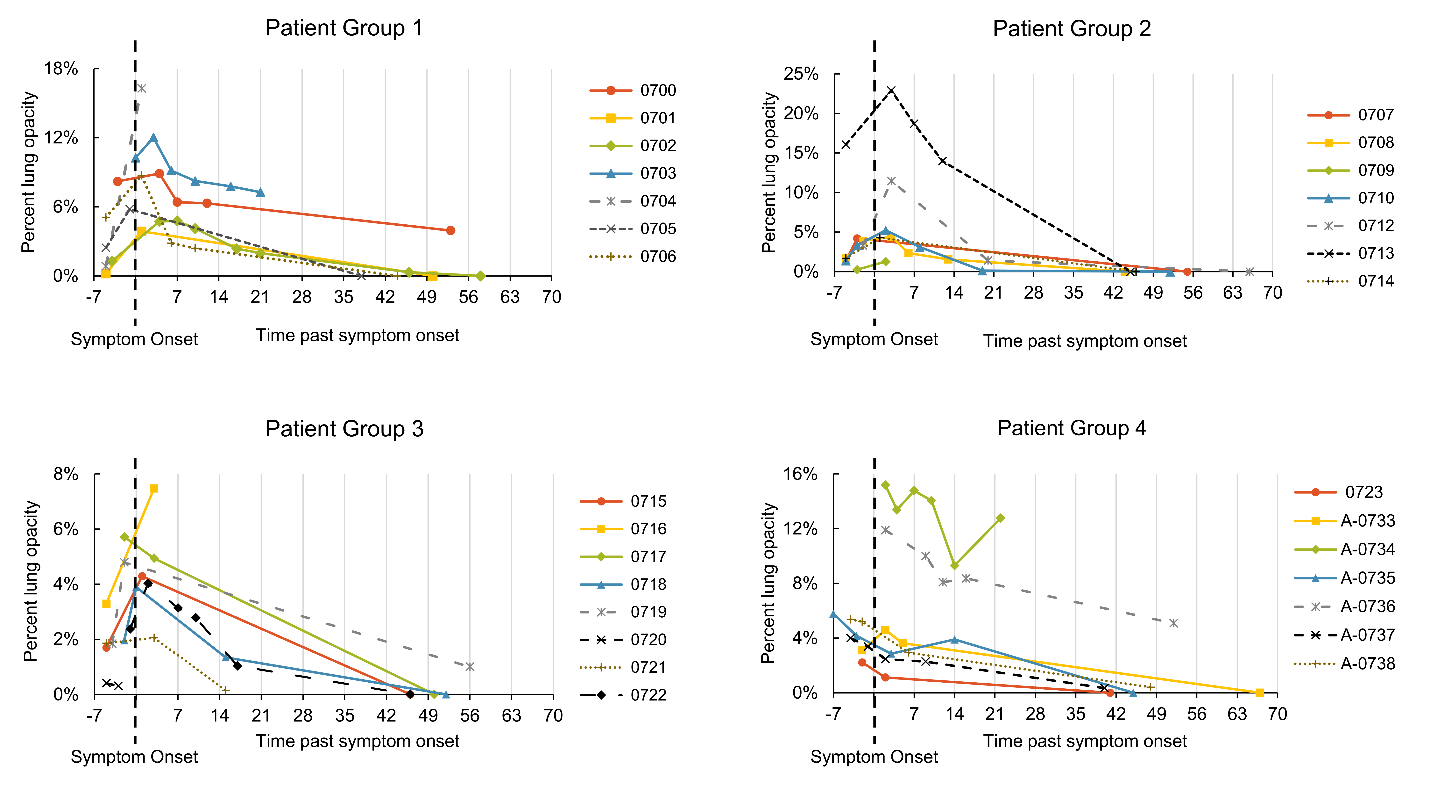


**Supplemental Figure S1.** Individual patient curves for percent lung opacity shown in four different and randomly subdivided patient groups so individual curves can be differentiated.


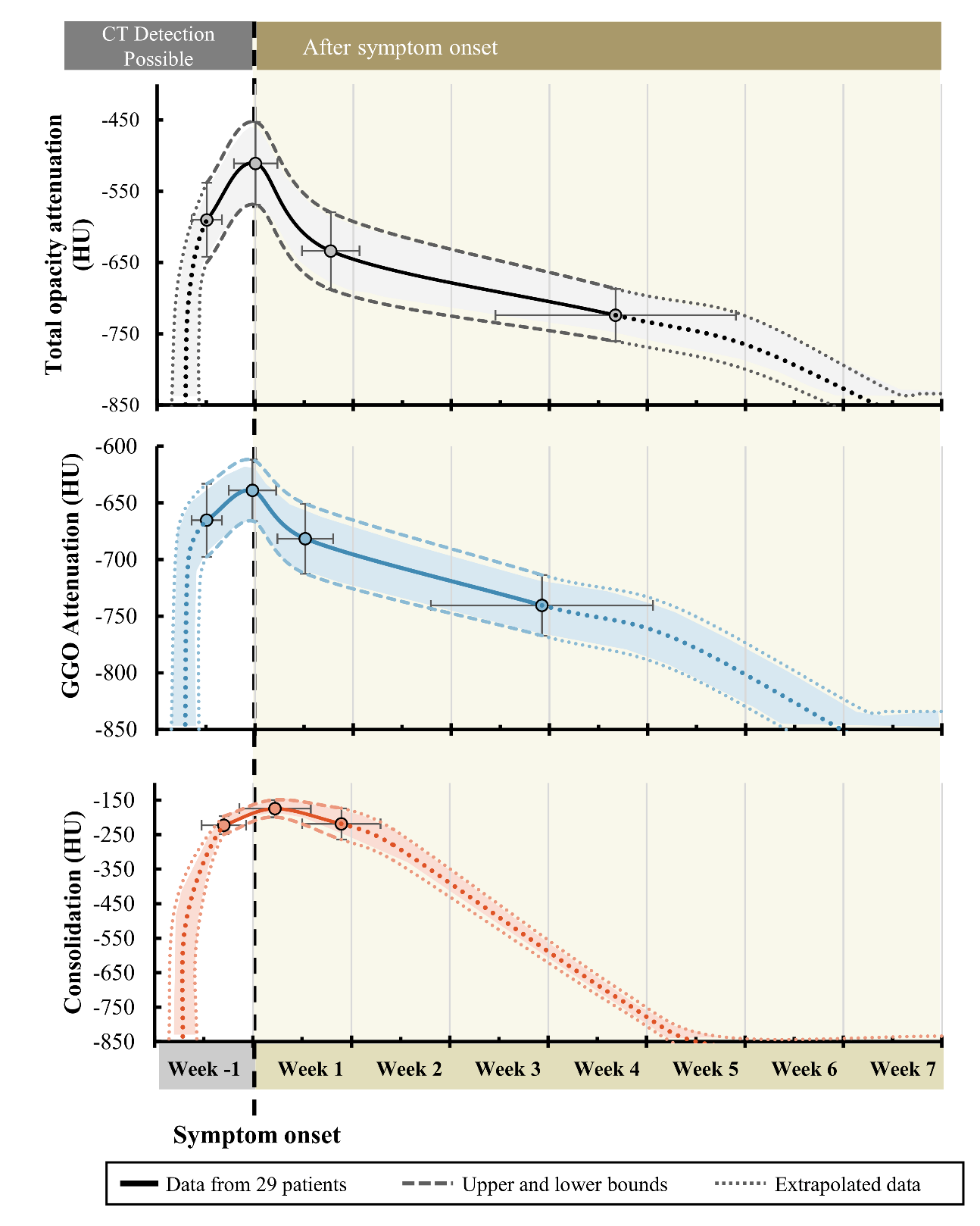


**Supplemental Figure S2.** Detailed curves showing COVID-19 lung opacity attenuation and subtypes, generated from 29 patients with sequential CTs. The upper and lower bounds and error bars represent the standard deviation of the data.


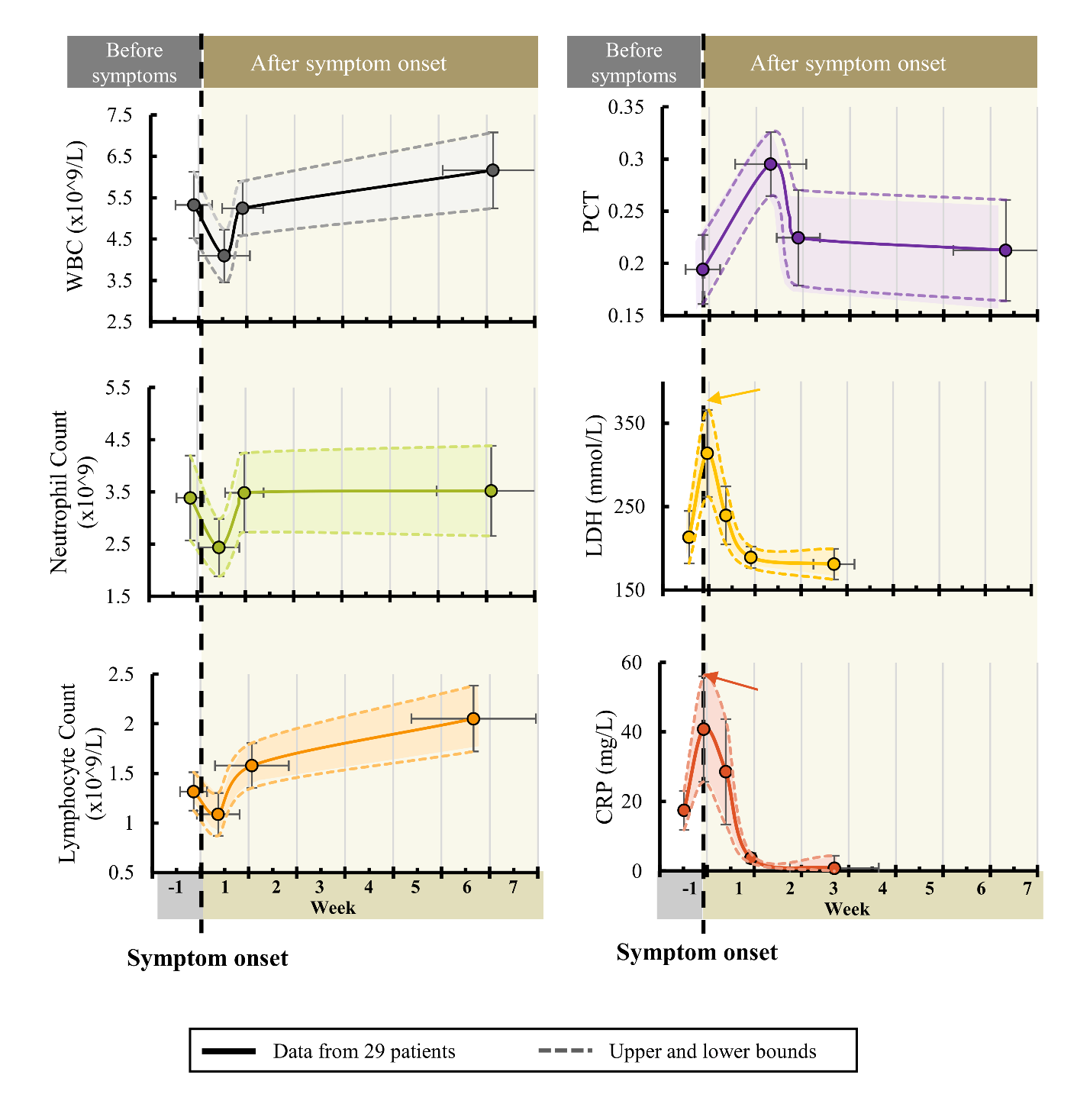


**Supplemental Figure S3.** Detailed curves with average and upper and lower bounds (standard deviation) of laboratory values.

**
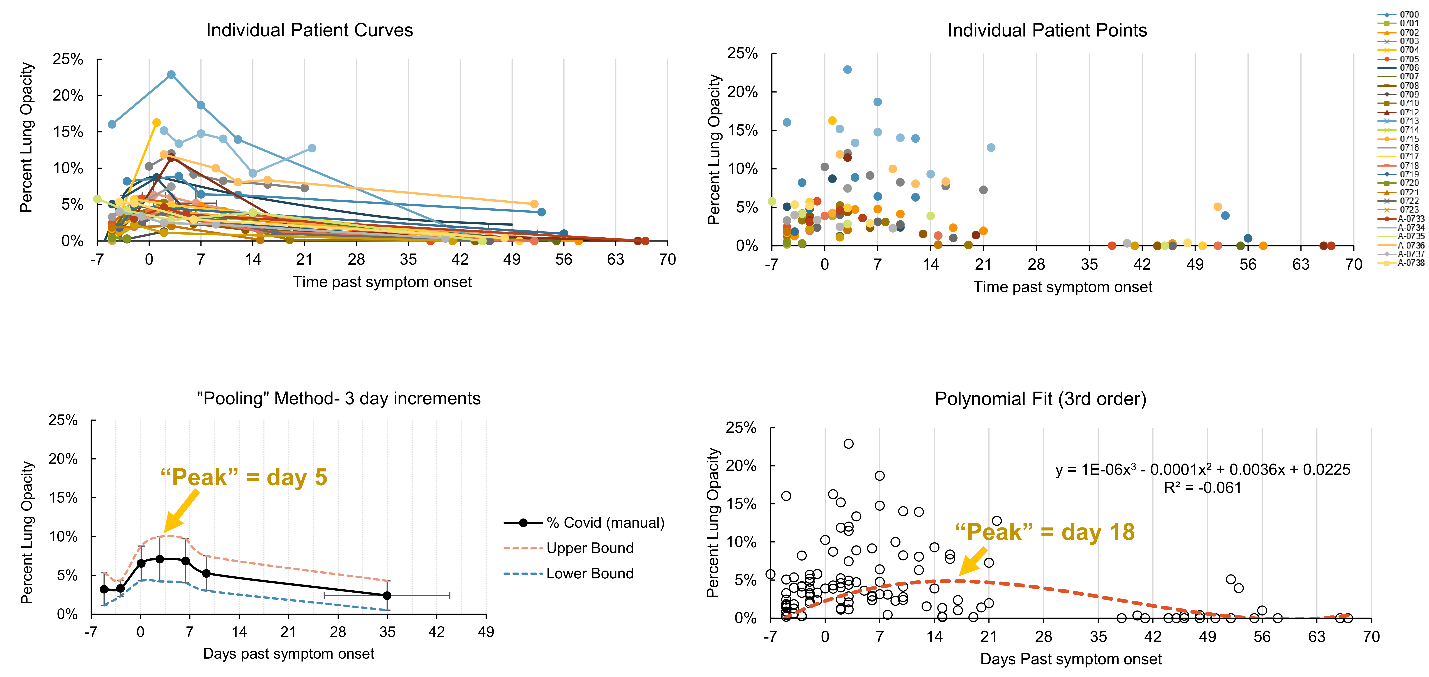
**

**Supplemental Figure S4. Application of different methodologies for investigation of COVID-19 disease dynamics. Top left-** Individual patient curves. Top right- individual patient points. Bottom left- a pooling method, where quantitative imaging results are pooled from 3-day increments. The results from this analysis suggest that peak lung opacity occurs 5 days after symptom onset. Bottom right- individual quantified points are collated and a polynomial curve is fit. This method indicates the peak lung opacity occurs at 18 days after symptom onset. Both methods analyzed quantified findings from CT scans autonomous of individual patient dynamics.

**Supplemental Video**. Dynamic updating of labs and imaging parameters in relation to generalized curves of mild to moderate disease could provide a framework for measurement of multi-parametric phenotypes during disease course
