# Supplementary figures and images for: Generalized chest CT and lab curves throughout the course of COVID-19
Source: Sci Rep. 2021 Mar 25;11:6940. doi: 10.1038/s41598-021-85694-5 (PMC7994835; doi:10.1038/s41598-021-85694-5)

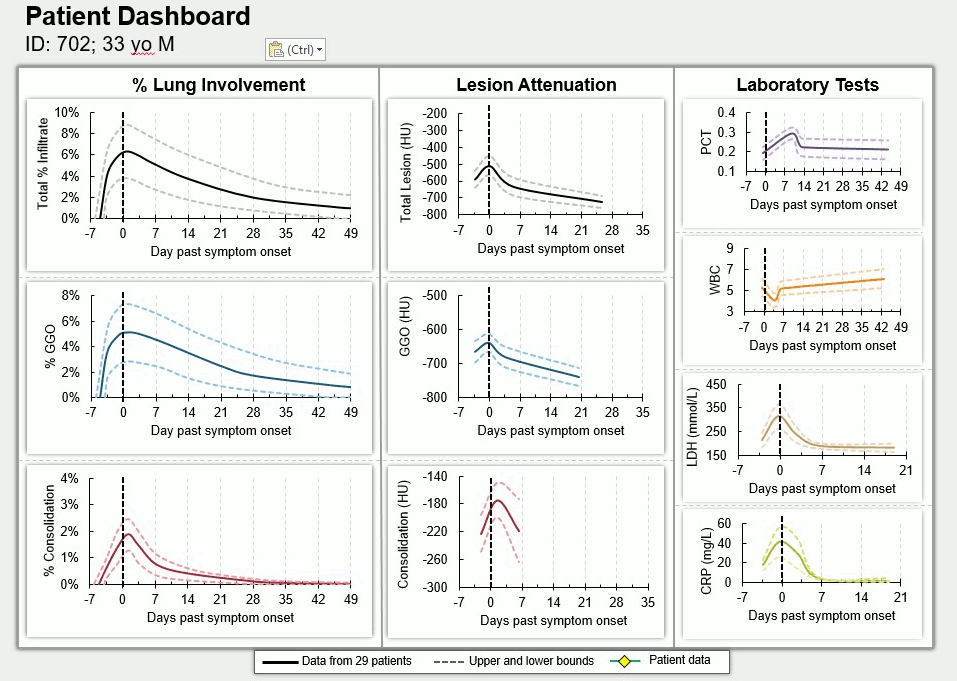

Supplement: Supplementary file 2 — Supplementary Video. [file 41598_2021_85694_MOESM2_ESM.gif]
